# Draft Genome Sequence and Annotation of Phyllosphere-Persisting *Salmonella enterica* subsp. *enterica* Serovar Livingstone Strain CKY-S4, Isolated from an Urban Lake in Regina, Canada

**DOI:** 10.1128/genomeA.00884-15

**Published:** 2015-08-13

**Authors:** Dinah D. Tambalo, Benjamin J. Perry, Stephen F. Fitzgerald, Andrew D. S. Cameron, Christopher K. Yost

**Affiliations:** University of Regina, Regina, Saskatchewan, Canada

## Abstract

Here, we report the first draft genome sequence of *Salmonella enterica* subsp. *enterica* serovar Livingstone. This *S*. Livingstone strain CKY-S4 displayed biofilm formation and cellulose production and could persist on lettuce. This genome may help the study of mechanisms by which enteric pathogens colonize food crops.

## GENOME ANNOUNCEMENT

*Salmonella* is a major cause of foodborne illness with a global burden estimated at 93.8 million cases annually (1). Fresh fruits and vegetables are recognized as important routes for transmission of foodborne pathogens (2–4). These pathogens can contaminate produce during production and handling. Following contamination, *Salmonella* has been shown to colonize and survive on vegetable crops (5–7).

*Salmonella enterica* subsp. *enterica* serovar Livingstone CKY-S4 was isolated from Wascana Lake, located in Regina, Saskatchewan, Canada. The strain was serotyped as serovar Livingstone at the Saskatchewan Disease Control Laboratory. Strains classified as Livingstone serotype have been isolated from water, poultry, and animal products (8), although human infections involving *Salmonella* Livingstone are sporadic (9). *S*. Livingstone CKY-S4 exhibited biofilm formation and cellulose production and was resistant to streptomycin, erythromycin, and spectinomycin. A plant colonization experiment measured the ability of CKY-S4 to persist on Romaine lettuce (cv. Paris Island), with a reduction rate of −0.31 log_10_ CFU per day. CKY-S4 persisted for at least 9 days with a major reduction of 4-log CFU/leaf, in the first 5 days.

Genomic DNA was isolated using a Bio Basic Canada DNA isolation kit, and prepared for Illumina sequencing using a NEBNext Ultra DNA library prep kit, with size selection for approximately 700-bp fragments of genomic DNA. DNA sequencing was performed using an Illumina MiSeq, with 300-bp paired-end version 3 chemistry. Raw fastq sequence data were filtered for PhiX sequence missed by the MiSeq filtering pipeline using Bowtie2 (10), and the remaining 5,076,916 paired-end reads were used for assembly.

Genome assembly was performed using the A5-MiSeq Assembly pipeline (11) and resulted in a consensus length of 4,803,211 bp composed of 31 contigs. The assembly had an *N*_50_ value of 445,170 bp, an average coverage of ×260, and a GC content of 52.1% and could have 75% of the genome represented by 7 large contigs. The annotation revealed 4,473 coding sequences within the genome. In addition, 48 pseudogenes, 15 noncoding RNAs (ncRNAs), and 2 clustered regularly interspaced short palindromic repeat (CRISPR) arrays were found.

Using the phage search tool (PHAST), three prophage-like regions were identified within the genome that shared homologous regions to phage BcepMu, SE1, and *Cronobacter* phage vB_CsaM_GAP32, with similarity scores of 20, 60, and 50, respectively (12). Antibiotic resistance genes were searched for using the antibiotic resistance genes database (ARDB). Multidrug efflux pump encoding genes *acrB*, *mdtK*, and *mdtL* were identified, as well as the macrolide-specific efflux pump *macB* and the penicillin binding protein *pbp2.* Further analysis of the annotation also confirmed the presence of the *Salmonella* pathogenicity islands required for invasion (SPI-1) and intracellular survival (SPI-2) within a mammalian host.

This is the first draft genome of a *Salmonella* strain in the serotype Livingstone. Its ability to colonize the phyllosphere of lettuce has been measured and its genome contains multiple antibiotic resistance genes, and three prophage-like regions. Availability of the draft genome increases the diversity of *Salmonella* genomes currently available for comparative analysis.

### Nucleotide sequence accession numbers.

The draft genome sequence of *Salmonella* Livingstone CKY-S4 was deposited in the GenBank/EMBL database with the accession number JZWK00000000. The version described in this paper is the first version, JZWK00000000.1.
